# Do natural landscapes reduce future discounting in humans?

**DOI:** 10.1098/rspb.2013.2295

**Published:** 2013-12-22

**Authors:** Arianne J. van der Wal, Hannah M. Schade, Lydia Krabbendam, Mark van Vugt

**Affiliations:** 1 Department of Social and Organizational Psychology, VU University, Amsterdam, The Netherlands; 2 Department of Educational Neuroscience, VU University, Amsterdam, The Netherlands

**Keywords:** temporal discounting, biophilia, life history, evolutionary psychology, nature, sustainability

## Abstract

An important barrier to enduring behavioural change is the human tendency to discount the future. Drawing on evolutionary theories of life history and biophilia, this study investigates whether exposure to natural versus urban landscapes affects people's temporal discount rates. The results of three studies, two laboratory experiments and a field study reveal that individual discount rates are systematically lower after people have been exposed to scenes of natural environments as opposed to urban environments. Further, this effect is owing to people placing more value on the future after nature exposure. The finding that nature exposure reduces future discounting—as opposed to exposure to urban environments—conveys important implications for a range of personal and collective outcomes including healthy lifestyles, sustainable resource use and population growth.

## 1. Introduction

An important barrier to fostering sustainable behavioural change is that humans have an evolved bias to prefer immediate rewards over long-term rewards [1–3]. This universal and surprisingly strong tendency to discount the future is a contributing factor to various individual and societal challenges, such as obesity, substance abuse, pollution, resource exploitation and overpopulation [4,5]. An important scientific question is whether people's discount rates vary, and if so why. Policy-makers require better knowledge about human temporal discount functions to devise effective strategies to improve public health and conserve natural resources.

### (a) Evolutionary principles behind temporal discounting

Evolutionary theories of life-history trade-offs suggest that organisms respond adaptively to environmental cues associated with the presence of threats and opportunities in their ecology [6]. Organisms adopt a slow reproductive strategy when resources are abundant and the environment is relatively benign and stable, whereas they adopt a fast reproductive strategy when there is competition for resources, and the environment is relatively hostile and unstable [7]. Animal studies show that discount rates are higher in response to environmental factors (e.g. food scarcity). For instance, pigeons at 80% of their body weight selected the smaller immediate food reward more often than at 95% of their body weight [8]. Human decision-making also varies predictably with ecological factors. In a study comparing different neighbourhoods within the same city (Chicago, IL), the median age of mothers giving birth was 22.6 years in neighbourhoods with a low life expectancy, whereas it was 27.3 years in neighbourhoods with a high life expectancy [9].

Environmental factors can affect the psychology of temporal decision-making more directly too. Temporal discounting is typically assessed by offering individuals choices between different monetary sums with different time intervals [10]. Although $100 is the same amount now or in one month's time, its value will be discounted when given with a delay. Individual differences in discount rates exist and they may be a function of socio-ecological factors. A recent study found that individuals who grew up in a poor and dangerous neighbourhood discounted the future more after they were exposed to mortality cues [11].

### (b) Environmental influences on temporal discounting

Here, we argue that cues associated with environmental uncertainty and resource competition affect future discounting in humans. Inspired by the biophilia hypothesis, which assumes that humans have an innately emotional affiliation to other living organisms [12,13], we believe that when people are being exposed to scenes of natural environments, as opposed to man-made, urban environments, this will reduce future discounting. Natural landscapes, especially lush ones, are intrinsically rewarding and enjoyable as they provide cues of predictability and resource abundance, at least for ancestral humans, whose psychology is likely to be still affecting modern humans [5]. By contrast, urban landscapes—which are entirely novel on an evolutionary time scale—are inherently unstable, and convey the perception of intense social competition among humans for all kinds of resources, such as status, goods and mates. As a consequence, we hypothesize that exposure to natural scenes will make people discount the future less, whereas exposure to urban scenes will be likely to have the opposite effect.

This finding is in line with studies showing the positive effects of nature exposure on self-control and prosociality. A US study shows that city children who live in homes near nature score higher on tests of concentration, impulse inhibition and delay of gratification [14]. Similarly, priming adults with scenes of natural beauty increases other-regarding preferences [15].

### (c) The psychology of temporal discounting

No recent research has looked directly at whether exposure to nature versus urban scenes inspires people to reduce future discounting, nor at its underlying proximate, psychological mechanisms. There are various possibilities. Peters & Büchel [16] and Figner *et al*. [17] show that temporal discounting decisions are influenced by two separate neural mechanisms having to do with either self-control or future valuation. There is evidence that exposure to nature increases self-control, as indicated by a study among inner urban children [14]. After watching a short video of plants growing, consumers exercised more self-control in purchasing behaviour [18]. In terms of valuing the future, several studies show that individuals become more environmentally aware after watching natural landscape scenes [15,19,20].

Thus, integrating evolutionary theories about life history and biophilia, our main hypothesis is that when people are exposed to scenes of natural landscapes their discount rates are lower compared with exposure to urban landscapes (Hypothesis 1). Further, this effect is expected to be mediated by either an increase in self-control (Hypothesis 2a), future reward valuation (Hypothesis 2b) or perhaps a combination (Hypothesis 2c) after nature exposure. We report the findings of two laboratory experiments and a field study that are consistent with our main hypothesis. Based on previous studies, we also explore whether these effects are being moderated by the amount of nature available in the area in which participants currently live or grew up [21].

## 2. Experiment 1

Experiment 1 tested Hypothesis 1: whether exposure to natural landscapes reduces future discounting compared with exposure to urban landscapes.

### (a) Method

Forty-seven participants (*M*_age_ = 20.23, s.d. = 2.16; 53.2% female), recruited through advertisements on posters in several university buildings, took part in the experiment. The standard protocol for this study (and the next) was as follows. Participants were welcomed by an experimental assistant, who was blind to the hypotheses, and randomly assigned by the order of arrival to the laboratory to either the nature or urban condition (between-subject design), which differed in the landscapes depicted on the photograph stimuli (see the electronic supplementary materials for the photographs). Per condition, three photographs were displayed on the computer screen, each for 2 min, accompanied by an audio script to encourage participants to ‘immerse themselves in the environment shown in the photograph’ [15]. Thereafter, participants completed a standard temporal discounting game [3]. Participants made seven binary intertemporal choices between two monetary options: 100 euros now or a larger sum that grew with 10-euro increments from 110 to 170 euros, after 90 days. These responses determined each individual's indifference point, the choice at which participants switch from selecting the smaller immediate reward to the larger delayed reward [22]. A choice for a lower delayed reward (i.e. a lower indifference point) indicates lower discounting. Participants were informed that they would be paid the money of one of the choices they made in the temporal discounting game through random selection. For reasons of convenience, this amount was directly paid out after the experiment, accompanied with a debriefing about the study's purpose. Finally, participants answered two manipulation check questions (‘How urban (natural) did you find the scenes in the photographs?’) and reported their demographics, including gender, age, and the postal codes of their former and current homes to calculate an index of the naturalness of the home environment, following the Netherlands Bureau of Statistics guidelines [23].

### (b) Results

If not mentioned otherwise, the analyses were conducted with the general linear model. Statistical assumptions of normal distribution as well as homogeneity were met. There were no differences between the conditions regarding age or gender and these did not influence discount rates (all *p* > 0.05). Nature photographs were indeed rated as more natural than the urban photographs (*M*_nature_ = 6.61, s.d. = 1.27 versus *M*_urban_ = 1.42, s.d. = 0.78), and less urban than the urban photographs (*M*_nature_ = 1.26, s.d. = 0.92 versus *M*_urban_ = 5.71, s.d. = 0.96). Hence, the manipulation seems to be effective.

**Figure 1.**
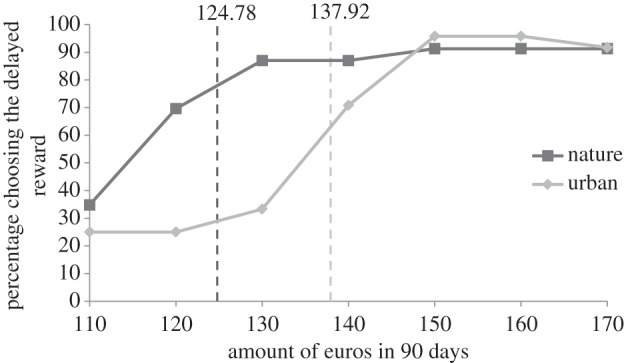
Percentage of participants that preferred the ‘*x*’ amount of euros in 90 days over the 100 euros now (Experiment 1), including the average individual indifference point for each condition. Nature condition differs significantly from the urban condition (*p* < 0.05).

Confirming the main hypothesis, the results of Experiment 1 showed that nature exposure significantly influenced temporal discounting in the predicted direction (*F*_1,45_ = 5.31, *p* = 0.026, part. **η**^2^ = 0.11). The individual indifference point—the point at which people switch to the larger delayed reward—was lower for participants in the nature condition compared with the participants in the urban condition (*M*_nature_ = 124.78, s.d. = 19.97 and *M*_urban_ = 137.92, s.d. = 19.11). Taken together, participants in the nature condition showed about a 10% lower temporal discount rate than participants in the urban condition (figure 1).

Regression analyses finally revealed that future discounting was not affected by the greenness of the area in which people either currently live in or grew up in (both *p* > 0.05).

## 3. Experiment 2

Experiment 2 aimed to find evidence for the proximate psychological mechanisms driving the difference between natural versus urban landscape exposure. Is the difference in discounting mediated by an increase in self-control, future reward valuation or perhaps a combination? We added a control condition without a photograph manipulation to examine whether either the nature or urban landscape manipulation was driving the effect on temporal discounting. We also used a different temporal discounting game.

### (a) Method

The same recruitment procedure was used as for Experiment 1. Sixty-seven participants (*M*_age_ = 20.03, s.d. = 1.83; 71.6% female) were randomly assigned (by the order of arrival to the laboratory by an assistant blind to the hypotheses) to either the nature, urban or control condition. Through checking their email addresses, it was ensured that none of the participants in the first study took part. Participants were primed by three nature or urban landscape photographs; in the control condition, no prime was administered. Participants completed a different temporal discounting game developed by Kirby *et al*. [24]. They made 18 intertemporal choices between two monetary options each: a specified sum now (ranging from 11 to 80 euros) or a larger sum (ranging from 25 to 85 euros) after a specified delay, ranging from 7 to 91 days. Choices were converted into a discount-rate parameter (*k*), ranging from 0.0025 to 0.25 (*k* = (future euro/now euro − 1)/delay (in days)) [24]. A lower discount-rate parameter indicates less temporal discounting.

We administered the standard Stroop colour-word test as a measure of self-control [25] and a future valuation task, in counterbalanced order. Participants completed 18 congruent and 18 incongruent trials of the Stroop colour-word test. A higher reaction time difference between the incongruent and the congruent trials indicates lower levels of self-control. In the valuation task, participants rated 18 single pay-offs from the Kirby *et al*. [24] temporal discounting game (e.g. 80 euros in 14 days) on a 100-point scale from very attractive (100) to very unattractive (0) [17]. Finally, participants answered the manipulation checks and demographic questions, and got paid the amount of one (randomly selected) choice they made in the game.

### (b) Results

The analyses were again conducted with the general linear model, and statistical assumptions of normal distribution and homogeneity were met. No differences were found for age or gender between conditions (both *p* > 0.05). Nature photographs were again rated more natural than the urban photographs (*M*_nature_ = 6.81, s.d. = 0.40 versus *M*_urban_ = 1.48, s.d. = 0.73), and less urban than the urban photographs (*M*_nature_ = 1.14, s.d. = 0.36 versus *M*_urban_ = 5.74, s.d. = 1.25).

**Figure 2.**
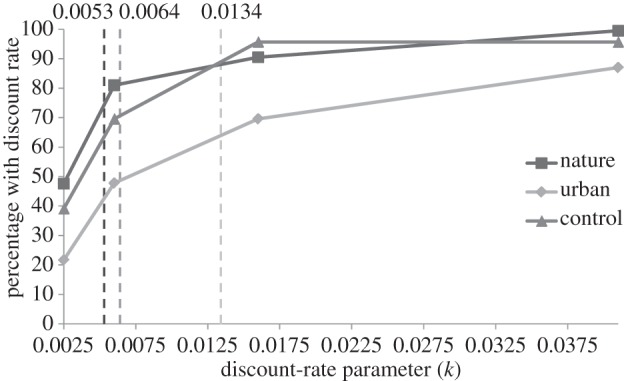
Percentage of participants that were indifferent at the different discount-rate parameters (*k*) (Experiment 2), including the average individual indifference point for each condition. Nature condition differs significantly from the urban condition (*p* < 0.05).

Hypothesis 1 was again confirmed. Condition affected temporal discounting (*F*_2,64_ = 3.69, *p* = 0.030, part. **η**^2^ = 0.10). *Post hoc* analyses showed that the individual indifference point in the nature condition (*M*_nature_ = 0.0053, s.d. = 0.0009) was lower than that in the urban condition (*M*_urban_ = 0.0134, s.d. = 0.0036; *p* = 0.012). This indicates that participants in the nature condition showed, on average, a 16% reduction in future discounting compared with the urban condition. The control condition fell in between the nature versus urban conditions (*M*_control_ = 0.0064, s.d. = 0.0014), yet these differences were not statistically significant (all *p* > 0.05; figure 2).

No overall effect was found of condition on future valuation (*F*_2,64_ = 2.01, *p* = 0.143); however, *post hoc* analyses suggest that participants in the nature condition (*M*_nature_ = 70.45, s.d. = 14.13) valued future rewards more than the control condition (*M*_control_ = 59.18, s.d. = 18.29; *p* = 0.049). The urban condition (*M*_urban_ = 64.40, s.d. = 22.23) did not differ significantly from the control condition (*p* > 0.05). This indicates that nature exposure increases future valuation, confirming Hypothesis 2b. Regression analysis showed that future valuation predicted temporal discounting (*b* = −0.001, *F*_1,65_ = 28.92, *p* < 0.001, part. **η**^2^ = 0.31). To establish whether future valuation mediates the effect of nature exposure on temporal discounting, indirect effects analysis by Preacher & Hayes [26] was conducted. For this analysis, the urban and control conditions were combined because they did not differ on discounting. The effect of condition on the discount-rate parameter was mediated by the valuation of the future rewards as predicted by Hypothesis 2b (*b* = −0.009, 95% CI [−0.029, −0.001]).

Analyses with regard to self-control revealed no effect across the three conditions (*F*_2,64_ = 2.36, *p* = 0.103). *Post hoc* analyses showed that participants in the nature condition (*M*_nature_ = −3.93 ms, s.d. = 18.11) had a lower reaction time difference between the incongruent and the congruent trials of the Stroop colour-word test, compared with the control condition (*M*_control_ = 50.41 ms, s.d. = 17.31; *p* = 0.034). However, this effect was completely driven by outliers (one participant in the nature condition showed a reaction time difference of −383.83 ms and three outliers in the control condition showed a reaction time difference greater than 225 ms). In addition, regression analysis showed that the performance on the Stroop colour-word test did not predict temporal discounting (*p* > 0.05). Thus, both Hypotheses 2a and 2c can be rejected. Finally, temporal discounting was not affected by whether people currently live or grew up in a green environment (both *p* > 0.05).

## 4. Experiment 3

Experiment 3 was a field study in which we examined whether the differences in temporal discounting also occurred when participants were asked to walk through either a real natural or urban landscape environment.

### (a) Method

Advertisements about the study were placed at grocery stores in the city of Amsterdam, The Netherlands, to recruit participants. Forty-three participants (*M*_age_ = 31.84, s.d. = 11.76; 60.5% female) took part and were randomly assigned (by the order of contacting us) to either the Amsterdam forest (nature condition) or to the Amsterdam Zuidas, which is a built-up area of Amsterdam (urban condition). Participants and experimental assistants (blind to the hypotheses) met at the location of the study. A map with directions was sent by email. Participants were asked to immerse themselves in the environment by walking through it by themselves for 5 min. Thereafter, participants sat down on a bench and received a table from the assistant to play the temporal discounting game, previously used by Wilson & Daly [3]. Future valuation was assessed with the same task as in the second study and self-control was being assessed with the state ego-depletion scale (**α** = 0.88) (J. M. Twenge, M. Muraven, D. M. Tice 2004, unpublished data). This scale contains 25 items (e.g. ‘Right now, it would take a lot of effort for me to concentrate on something’) rated on a 7-point scale, from strongly disagree (1) to strongly agree (7). We also administered a mood scale (**α** = 0.76) [27]. The scale contained 16 items (e.g. ‘I feel jittery’, ‘I feel happy’; 1 = strongly disagree, 7 = strongly agree). Finally, participants reported their demographics and got paid according to one of the (randomly selected) intertemporal choices they made in the decision task.

### (b) Results

Analyses were again performed with the general linear model, and the statistical assumptions of normality and homogeneity of data were met. There were no demographic differences between conditions (*p* > 0.05). Participants in the nature condition reported a greater positive mood than participants in the urban condition (*F*_1,41_ = 4.54, *p* = 0.042, part. **η**^2^ = 0.14), with *M*_nature_ = 5.53 (s.d. = 0.40) and *M*_urban_ = 5.14 (s.d. = 0.60). However, mood did not affect temporal discounting (*p* > 0.05).

**Figure 3.**
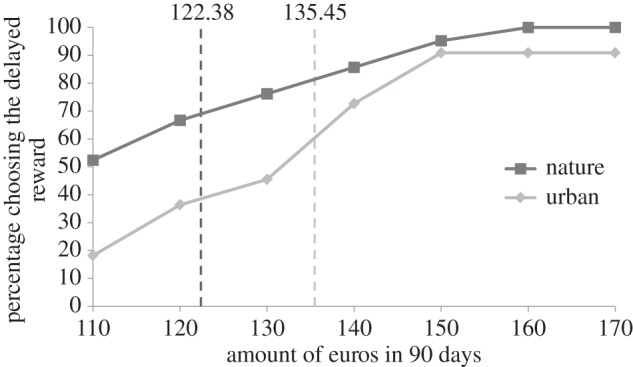
Percentage of participants that preferred the ‘*x*’ amount of euros in 90 days over the 100 euros now (Experiment 3), including the average individual indifference point for each condition. Nature condition differs significantly from the urban condition (*p* < 0.05).

Confirming Hypothesis 1, participants in the nature versus urban condition showed a significant difference in temporal discounting (*F*_1,41_ = 5.41, *p* = 0.025, part. **η**^2^ = 0.12). In the nature condition, the individual indifference point of the participants was lower than that in the urban condition, with *M*_nature_ = 122.38 (s.d. = 16.40) and *M*_urban_ = 135.45 (s.d. = 20.17). On average, we found a 10% reduction in future discounting in the nature condition versus urban condition (figure 3).

Confirming Hypothesis 2b, participants in the nature condition valued future rewards more than that in the urban condition (*F*_1,41_ = 7.12, *p* = 0.011, part. **η**^2^ = 0.15), with *M*_nature_ = 91.62 (s.d. = 13.25) and *M*_urban_ = 73.82 (s.d. = 27.68). Regression analyses showed that future valuation predicted temporal discounting (*b* = −0.41, *F*_1,41_ = 13.17, *p* = 0.001, part. **η**^2^ = 0.24). The indirect effects analyses by Preacher & Hayes [26] revealed that future valuation mediated the relationship between nature (versus urban) exposure and temporal discounting (*b* = −6.26, 95% CI [−16.26, −0.38]). No effect of condition on self-control was found (*p* > 0.05), nor did self-control predict temporal discounting (*p* > 0.05). Finally, regression analyses showed that temporal discounting was not affected by whether people grew up or currently lived in a rural or urban environment (both *p* > 0.05).

## 5. Discussion

All three studies, including a fairly realistic field study, showed that exposure to natural landscapes decreases temporal discounting and makes people care more for the future, with discount rates being 10–16% lower after nature exposure than after exposure to urban landscapes. Thus, cues of natural environments—as opposed to man-made urban environments—entice people to prefer greater delayed rewards over smaller immediate rewards. This is an important result because delay of gratification is an essential ingredient for promoting individual and social change pertaining to, for instance, healthy lifestyles, antisocial behaviour, resource conservation and population growth [5]. The results show further that, at the proximate psychological level, the beneficial effects of nature are mainly owing to people caring more about the future rather than a greater self-control or better mood. This is consistent with research showing that scenes of nature increase people's environmental awareness [19,20]. In terms of ultimate evolutionary explanations, our findings could be interpreted in terms of life-history trade-offs. Urban landscapes are inherently unpredictable as they convey intense social competition for status, goods and mates, and so they may entice people—either consciously or subconsciously—to adopt a faster life history. By contrast, nature exposure may encourage individuals to adopt a slower life-history strategy, perhaps because natural environments convey an abundance of natural resources, and hence less competition. This explanation is further consistent with the biophilia hypothesis by revealing the beneficial effects of nature exposure on short- and long-term personal well-being [12,13].

We should note various limitations of our research. First, our studies only used photographs of lush, green landscapes and it would be interesting to include dry, barren nature scenes in further research too. Future studies could also look at the impact of natural scenes with differing degrees of biodiversity on temporal discounting. Second, employing a within-subject (rather than a between-subject) design would have been stronger for detecting individual fluctuations in discount rates as a result of the manipulations, yet at the risk of participants guessing the study's predictions. A within-subject design could also have revealed whether the effects we found are owing to a reduced temporal discounting after nature exposure or an increased temporal discounting after urban exposure. The inclusion of a control condition was not conclusive, but the future valuation results suggest that nature exposure was driving the discounting difference. Finally, we examined various proximate psychological mediators of the nature-discounting effect, including mood, self-control and future valuation, but only found a mediating effect for the latter. Before dismissing the role of mood or psychological self-control, it would be useful to include better measures, such as the PANAS (for mood) [28] and the Tower Task (for self-control) [29]. A final suggestion for future research would be to add neuroscience measures, such as ERP and fMRI, to look more closely at the neural correlates of nature versus urban exposure on temporal discounting [21].

## 6. Conclusion

Many of the social and environmental problems the world faces nowadays, such as poverty, substance abuse, overpopulation and resource exploitation, are caused by citizens—and sometimes governments—adopting short-term decision-making strategies [5,30]. Our main finding suggests that exposing people to natural landscapes extends their time horizons, whereas exposure to urban landscapes narrows people's time perspectives. With the majority of people in the world now living in towns and cities, it may be important to find ways to unleash people's innate biophilia [12].
